# CD73-Adenosine A_1_R Axis Regulates the Activation and Apoptosis of Hepatic Stellate Cells Through the PLC-IP_3_-Ca^2+^/DAG-PKC Signaling Pathway

**DOI:** 10.3389/fphar.2022.922885

**Published:** 2022-06-16

**Authors:** Zhenni Liu, Xue Wu, Qi Wang, Zixuan Li, Xueqi Liu, Xiaodong Sheng, Hong Zhu, Mengda Zhang, Junrui Xu, Xiaowen Feng, Baoming Wu, Xiongwen Lv

**Affiliations:** ^1^ Inflammation and Immune Mediated Diseases Laboratory of Anhui Province, Anhui Institute of Innovative Drugs, School of Pharmacy, Anhui Medical University, Hefei, China; ^2^ Institute for Liver Diseases of Anhui Medical University, Hefei, China; ^3^ Seventh Affiliated Hospital of Sun Yat-Sen University, Shenzhen, China; ^4^ General Thoracic Surgery, The First Affiliated Hospital of Anhui Medical University, Hefei, China

**Keywords:** CD73, adenosine A1 receptor, alcohol-related liver fibrosis, apoptosis, PLC-IP_3_-Ca^2+^/DAG-PKC signaling pathway

## Abstract

Alcohol-related liver fibrosis (ALF) is a form of alcohol-related liver disease (ALD) that generally occurs in response to heavy long-term drinking. Ecto-5′-nucleotidase (NT5E), also known as CD73, is a cytomembrane protein linked to the cell membrane *via* a GPI anchor that regulates the conversion of extracellular ATP to adenosine. Adenosine and its receptors are important regulators of the cellular response. Previous studies showed that CD73 and adenosine A1 receptor (A_1_R) were important in alcohol-related liver disease, however the exact mechanism is unclear. The aim of this study was to elucidate the role and mechanism of the CD73-A_1_R axis in both a murine model of alcohol and carbon tetrachloride (CCl_4_) induced ALF and in an *in vitro* model of fibrosis induced by acetaldehyde. The degree of liver injury was determined by measuring serum AST and ALT levels, H & E staining, and Masson’s trichrome staining. The expression levels of fibrosis indicators and PLC-IP_3_-Ca^2+^/DAG-PKC signaling pathway were detected by quantitative real-time PCR, western blotting, ELISA, and calcium assay. Hepatic stellate cell (HSC) apoptosis was detected using the Annexin V-FITC/PI cell apoptosis detection kit. Knockdown of CD73 significantly attenuated the accumulation of α-SMA and COL1a1 damaged the histological architecture of the mouse liver induced by alcohol and CCl_4_. *In vitro*, CD73 inhibition attenuated acetaldehyde-induced fibrosis and downregulated A_1_R expression in HSC-T6 cells. Inhibition of CD73/A_1_R downregulated the expression of the PLC-IP_3_-Ca^2+^/DAG-PKC signaling pathway. In addition, silencing of CD73/A_1_R promoted apoptosis in HSC-T6 cells. In conclusion, the CD73-A_1_R axis can regulate the activation and apoptosis of HSCs through the PLC-IP_3_-Ca^2+^/DAG-PKC signaling pathway.

## Introduction

A grim outlook in the prevalence and mortality caused by ALD was projected by a recent Markov model for the next twenty years. Julien et al. (2020) ALD can be divided into simple fatty liver disease, alcohol-related hepatitis, liver fibrosis and cirrhosis (Li and Fan, 2018) The development of these diseases is caused mainly by the production of metabolic factors induced by alcohol. These metabolic factors can lead to lipid metabolism disorders, apoptosis and regeneration of liver cells, oxidative stress, collagen deposition, and disorders in the immune responses of the liver. The liver is one of the most important metabolic organs of the human body. If excess energy accumulates in the body, the storage capacity of the subcutaneous white adipose tissue is exceeded, and ectopic lipid deposition occurs in the liver, kidneys, and skeletal muscles. Neeland et al. (2016) Excessive intake of alcohol may not only lead to uncontrolled metabolism, but also destroy the beneficial effects on the cardiovascular system, resulting in metabolic syndrome. Wiel (2010); Pohanka (2016) Recent studies have demonstrated that alcohol can cause changes in the metabolism of mice after liver damage, including changes in the purine signaling pathway. Ma et al. (2020a).

Hepatic stellate cells (HSCs) are the main source of extracellular matrix (ECM), and excessive accumulation of ECM in the liver can lead to liver fibrosis. Zhang et al. (2018) Therefore, promoting the apoptosis of activated HSCs is considered a key step in the reversion of liver fibrosis Mederacke et al. (2013); Ding et al. (2019).

Ecto-5′-nucleotidase (NT5E), also known as CD73, is an ectomembrane protein linked to the cell membrane *via* a GPI anchor that regulates the conversion of extracellular ATP to adenosine. Ogata et al. (1990); Knofel and Strater (2001) Several studies have shown that CD73 is a key regulator in numerous pathophysiological processes, including immune homeostasis as it regulates the balance between pro-inflammatory ATP and immunosuppressive adenosine to prevent excessive immune responses. Bynoe et al. (2013); Xu et al. (2018) CD73 has also been shown to be involved in liver fibrosis, liver cancer, and other diseases. Allard et al. (2016); Ma et al. (2020b) Adenosine, a key endogenous molecule, is involved in several physiological and pathological processes by binding and activating the G protein-coupled adenosine receptor (AR). Faas et al. (2017) The AR has four subtypes: A_1_R, A_2A_R, A_2B_R, and A_3_R. The A_1_R can activate phospholipase C (PLC) through its Gq coupled-protein, by increasing the inositol 1,4,5- triphosphate (IP_3_) and diacylglycerol (DAG) content to regulate the release of Ca^2+^. Alzayady et al. (2016) Protein kinase C (PKC) is activated when DAG and Ca^2+^ act together, leading to the phosphorylation of its target proteins to achieve the desired biological effect. Adenosine receptors are widely present in HSCs, and in the process of ethanol metabolism, CD73 mediated adenosine production can activate AR. Activation of AR effects the development of alcohol-related fatty liver and liver fibrosis. Peng et al. (2009); Yamaguchi et al. (2017); Fausther (2018) Studies have shown that knockdown of A_1_R expression increased lipolysis. Johansson et al. (2008) In the formation of ALF, the cAMP and phosphoinositide (PI) signaling pathways, mediated by G protein-coupled receptors, are the main cell targets. The key signaling molecules of the PI pathway, DAG and PKC, are also important in the regulation of cell proliferation. Marinissena and Gutkind (2001); New and Wong (2007); Obeng et al. (2020).

Our previous study showed that the inhibition of CD73 promoted HSC-T6 cell apoptosis, however the specific mechanism was not clear. In addition, A_1_R is related to HSC-T6 cell activation, however its specific mechanism requires further investigation. Thus, we hypothesized that the CD73/A_1_R axis regulates HSC-T6 cell activation and apoptosis through the PLC-IP_3_-Ca^2+^/DAG-PKC signaling pathway.

## Materials and Methods

### Reagents

CPA (A_1_R agonist), DPCPX (A_1_R antagonist), UTP (IP_3_ precursor) and NECA (non-selective adenosine receptor agonist) were purchased from Tocris. Acetaldehyde was purchased from Damao. The primers were purchased from Jierui and Jeneral Biol. Antibodies against α-SMA, β-actin, PKC, and PLC were purchased from Bioss. Antibodies against A_1_R, COL1a1, Bax, Bcl-2, and cleaved-Caspase3 were purchased from Abcam. Antibodies against CD73 was purchased from Proteintect.

### Establishment of an Alcohol-Related Liver Fibrosis Model *in vivo*

C57BL/6J CD73 knock out (KO) mice used in our study were purchased from Cyagen Biosciences Inc. Mice were kept in SPF environment. Animals were serviced following the Guides of Center for Developmental Biology, Anhui Medical University for the Care and Use of Laboratory. 8–12 weeks male mice were used as an *in vivo* ALF model. In conclusion, after one week of adaptive feeding, 5% alcohol liquid diet was given for 8 weeks. High-concentration alcohol (5 g/kg) was administered by gavage twice weekly, and 10% CCl4 injections (1 ml/kg) were administered twice weekly for the last two weeks. The mice in the control group were injected with an equivalent volume of normal saline. Fasting for 9 h after the last injection, blood samples were collected, and related indicators were tested (Supplementary Figure S1). Ramirez et al. (2017).

### Alanine Aminotransferase/Aspartate Aminotransferase Activity

Serum isolated from the mice was centrifuged at 3,000 g for 30 min, and then tested with ALT and AST kits respectively. An alanine aminotransferase (ALT) assay kit and aspartate aminotransferase (AST) assay kit were obtained from Nanjing Jiancheng Bioengineering Institute (Nanjing, China).

### Histological Evaluation and Immunohistochemistry

After removing the liver tissue from the mouse, part of the liver tissue was fixed with formalin for approximately 24 h, and the remaining part was stored at −80°C. Paraffin-embedded tissue sections were 5-μm-thick sections, stained with hematoxylin and eosin (H and E), Oil Red O staining, Masson staining, Sirius red staining and immunohistochemical staining for CD73 (1:500, Proteintect) and A_1_R (1:100, Bioworld). Slides were scanned by an automatic digital slide scanner (Pannoramic MIDI, 3DHISTECH, Hungary).

### Cell Culture and Cell Model

The rat HSC-T6 cell line was obtained from Nanjing Kaiji Gene Biological Corporation (Nanjing, China). Cells were cultured in standard tissue culture plastic flasks in DMEM (Gibco, United States) with 10% FBS (Sijiqing, China) and penicillin/streptomycin (100 U/ml and 100 μg/ml respectively). The cells were incubated at 37°C, 5% CO_2_. Cells were treated with 200 µM acetaldehyde (Damao, China) to establish an alcohol related liver fibrosis cell model for 48 h Jia et al. (2020).

### Quantitative Real-Time PCR

Total RNA was extracted from HSCs with TRIzol reagent (Invitrogen, United States). The First-stand cDNA was synthesized from total with AMV Reverse Transcriptase according to the manufacturer’s protocol. Quantitative real-time PCR analyses for mRNA of A_1_R, α-SMA, COL1a1, β-actin were performed by PIKO REAL RT-PCR kits (Thermo Scientific, United States) using the following primer sequences: A_1_R forward: GCG AGT TCG AGA AGG TCA TC and reverse: GCT GCT TGC GGA TTA GGT AG, α-SMA forward: CGA AGC GCA GAG CAA GAG A and reverse: CAT GTC GTC CCA GTT GGT GAT, COL1a1 forward: GAT CCT GCC GAT GTC GCT AT and reverse: TGT AGG CTA CGC TGT TCT TGC A, β-actin forward: ACC ACA GCT GAG AGG GAA ATC G and reverse: AGA GGT CTT TAC GGA TGT CAA CG. CD73 forward: GGC AGA TGC TCT TCA CAA GG and reverse: CCT TCC AGA AGG ACC CTG TT. PCR was performed at 95°C for 10 min, followed by 40 cycles of amplification at 95°C for 15 s, 60°C for 30 s and 72°C for 30 s with PIKO REAL 96. The mRNA level of β-actin was used as an internal control and the fold-change for mRNA relative to β-actin was analyzed by the 2−^△△Ct^ method. PCR was performed in triplicate from three independent RNA samples and repeated at least three times.

### RNA Interference Analysis

SiRNA oligonucleotides against CD73 genes or scrambled sequences were as follows: CD73 siRNA (sense: 5′-GGU UGA GUU UGA UGA UAA A-3′ and antisense: 5′-GGU UGA GUU UGA UGA UAA A-3′); si-control with scrambled sequence (sense: 5′-UUC UCC GAA CGU GUC ACG U--3′ and antisense: 5′-ACG UGA CAC GUU CGG AGA A--3′). SiRNA oligonucleotides against A_1_R genes or scrambled sequences were as follows: A_1_R siRNA (sense: 5′- CCA GCA UUC UGA UCU ACA UTT -3′ and antisense: 5′- AUG UAG AUC AGA AUG CUG GTT -3′); si-control with scrambled sequence (sense: 5′- UUC UCC GAA CGU GUC ACG UTT -3′ and antisense: 5′- ACG UGA CAC GUU CGG AGA ATT -3′). HSC-T6 cell (1 × 10^5^) were cultured in 6-well plates for 24 h with antibiotic-free DMEM, and then according to the manufacturer’s protocol, Lipofectamine^TM^ 2000 (Invitrogen, United States) was used to transfect siRNA into cells. All transfection experiments were performed three times independently.

### Calcium Assay

HSC-T6 cells were incubated in a 15 mm^2^ cell culture flask with nonantibiotic DMEM overnight, Hank’s wash was used twice, and then the cells were incubated with Fluo-3/AM (4 µM) and Pluronic F-127 (0.02%) at 37°C for 30 min. After washing three-times with Hank’s solution to remove the extracellular Fluo-3/AM, cells were placed onto the stage of confocal laser scanning fluorescence microscopy and analyzed by Leica-sp5 LAS AF software.

### Enzyme-Linked Immunosorbent Assay of Diacylglycerol and IP_3_

The DAG and IP_3_ levels were measured by DAG and IP_3_ ELISA kits (R & D Systems, United States), respectively, according to the manufacturer’s instructions.

### Western Blot Analysis

Total protein was extracted from the tissue or cells with RIPA lysis solution (Beyotime, Shanghai, China) containing 1% PMSF and lysed on ice for 15 min, and then centrifuged at 12,000 g for 30 min at 4°C. Protein concentration was determined with a BCA protein assay kit (Boster, China). Total proteins were separated by SDS-PAGE and transferred onto a PVDF membrane. After blocking, nitrocellulose blots were incubated overnight with primary antibody at 4°C and then horseradish peroxidase conjugated with secondary antibody. Proteins were visualized with an ECL-chemiluminescent kit (Thermo Scientific, United States). The experiment was repeated at least three times from three independent samples.

### Flow Cytometry Analysis

An Annexin-V-FITC/PI apoptosis detection kit (Best Bio, China) was used to detect apoptosis. Cells (3 × 10^5^/ml) were transfected and seeded in a 6-well plate with CD73 siRNA/A_1_R siRNA. After the transfection, collect the cells in a 1.5 ml centrifuge tube, wash with PBS and add 400 µl Annexin binding solution to resuspend the cells, and then add 5 µl AnnexinV-FITC for staining. The solution was incubated together for 15 min, and the whole process was protected from light. Finally, 5 µl of PI was added and incubated for another 5 min, and a flow cytometer was used for detection.

### Statistical Analysis

All results are expressed as the mean ± SE using GraphPad Prism (San Diego, CA). Statistical significance was determined by either Student’s t-test for the comparison of the means or One-Way ANOVA (LSD). If *p* < 0.05, the difference was considered to be statistically significant.

## Results

### CD73 and A1 Receptor was Increased in Alcohol-Related Fibrotic Liver Tissues and Acetaldehyde-Induced Hepatic Stellate Cell-T6 Cell

**FIGURE 1:**
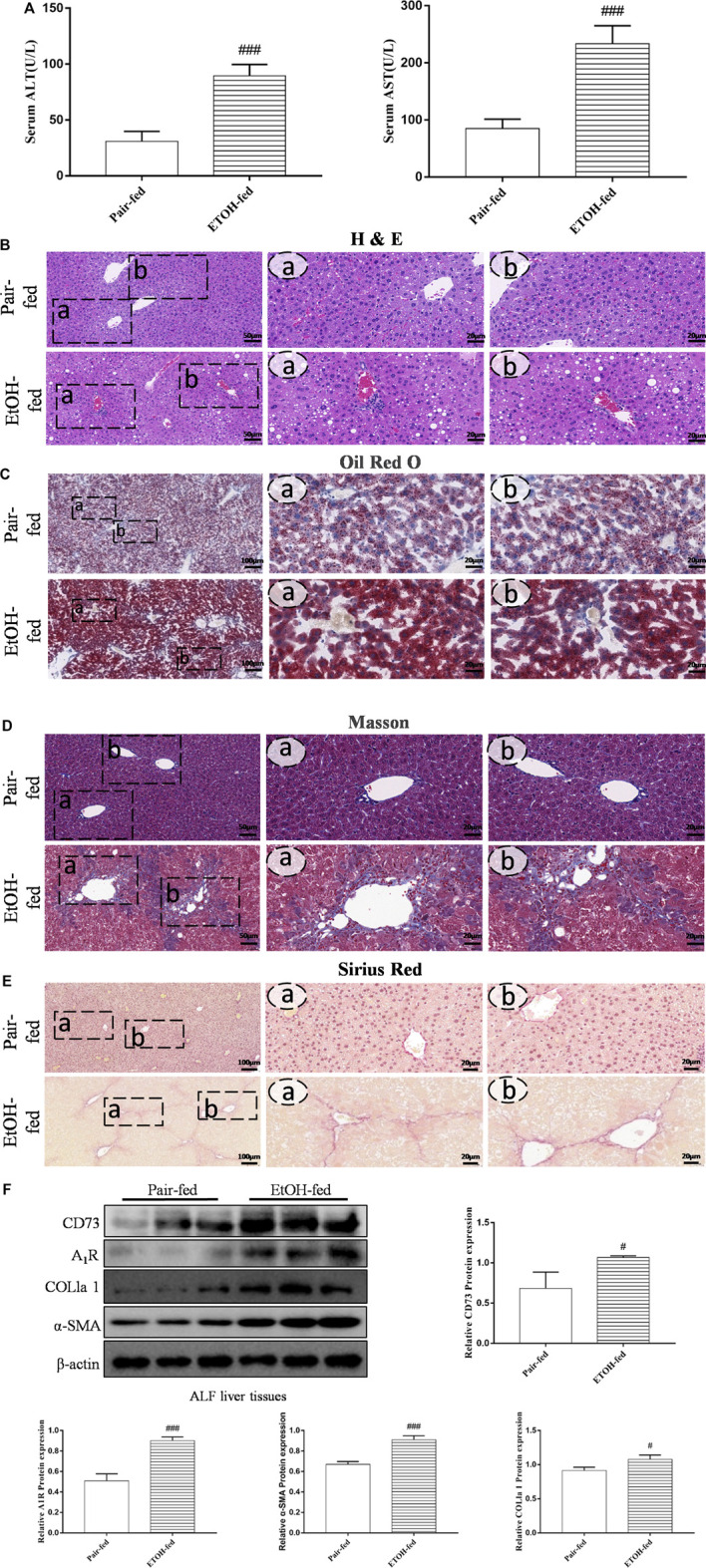
(Continued). CD73 and A_1_R was increased in alcohol-related fibrotic liver tissues. **(A)** Serum ALT and AST levels. **(B)** Representative HE staining of liver sections (50 μm). **(C)** Representative Oil Red O staining of liver sections (100 μm). **(D)** Representative Masson staining of liver sections (50 μm). **(E)** Representative Sirius Red staining of liver sections (100 μm). **(F)** The protein levels of CD73, A_1_R, α-SMA and COL1a1 in the liver. ^*#*^ *p* < 0.05, ^*###*^ *p* < 0.001 *vs.* the pair-fed group.

**FIGURE 2:**
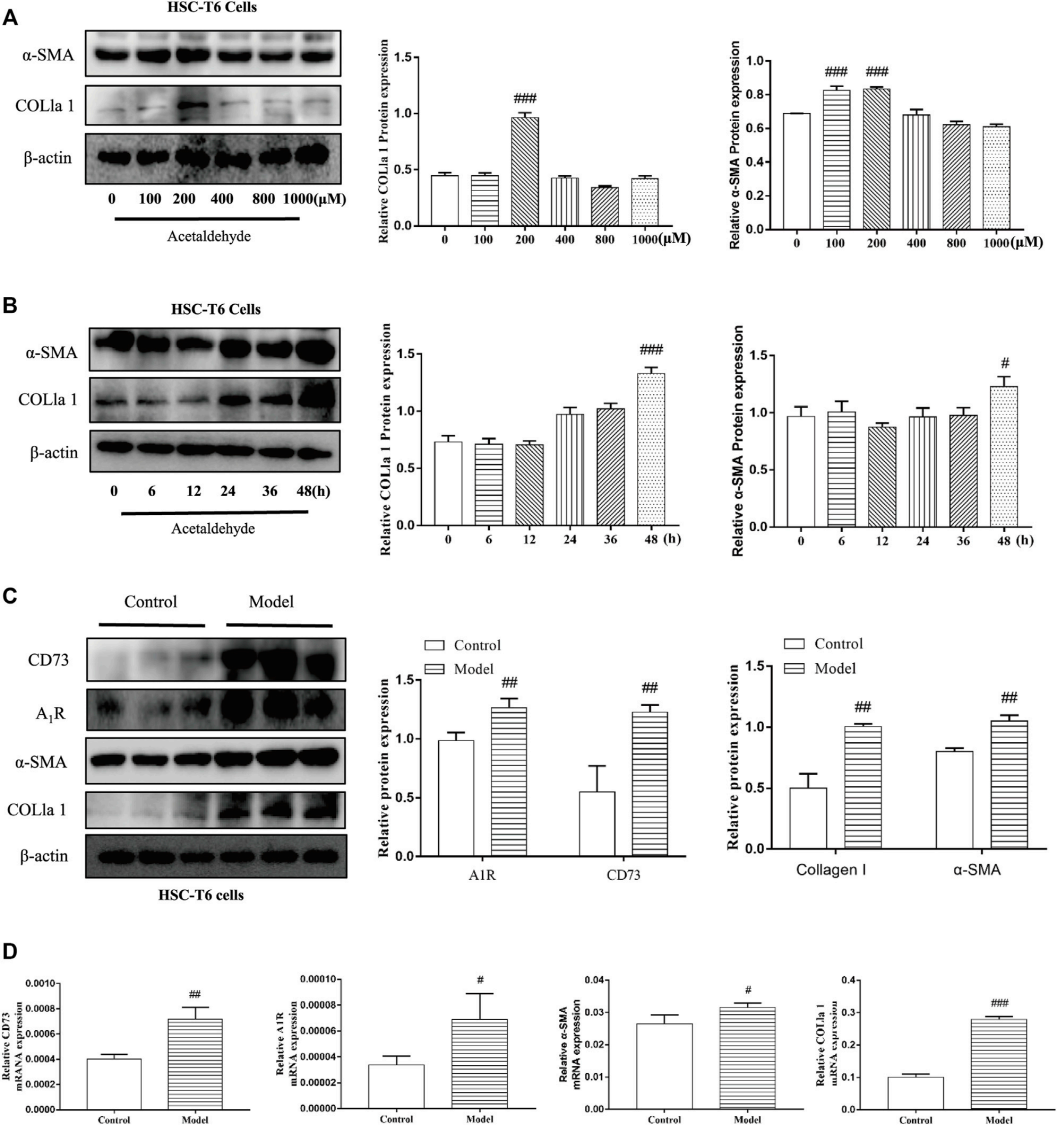
CD73 and A_1_R was increased in acetaldehyde-induced HSC-T6 cell. **(A,B)** The protein levels of α-SMA and COL1a1 in HSC-T6 cell. **(C)** The protein levels of CD73, A_1_R, α-SMA, and COL1a1 in HSC-T6 cell. **(D)** The mRNA levels of CD73, A_1_R, α-SMA, and COL1a1 in HSC-T6 cell. ^*#*^ *p* < 0.05, ^*##*^ *p* < 0.01, ^*###*^ *p* < 0.001 *vs.* the control group.

To determine whether CD73-A_1_R axis participates in alcohol-related liver fibrosis, we first established an alcohol-related liver fibrosis model in mice. As shown in Figure 1A, ALT and AST levels in the EtOH-fed group were significantly higher than those in the pair-fed group. H and E, Oil Red O, Masson staining and Sirius red staining were used to show liver tissue damage and lipid accumulation. As shown in Figures 1B,C, the EtOH-fed group showed abnormal liver cell cords and a large number of fat vacuoles and lipid accumulation compared with the control group. Masson and Sirius Red staining showed that collagen deposition increased (Figures 1D,E). At the same time, the Western blot results showed that the expression of α-SMA and COL1a1 were increased (Figure 1F). These indicated that the ALF model is successful.^25^ Based on the above model, we detected the expression of CD73 and A_1_R in liver tissue by Western blot. The results showed that the expression of CD73 and A_1_R in the EtOH-fed group was significantly upregulated (Figure 1F). We screened the right dose of acetaldehyde in HSC-T6 cell by Western blot. The Western blot results identified that the expression of α-SMA and COL1a1 reached the highest level after stimulation with 200 μM acetaldehyde for 48 h (Figures 2A,B). Then, we detected the expression of CD73 and A_1_R in HSC-T6 cell induced by acetaldehyde, which was consistent with the experimental results *in vivo*. The protein and mRNA levels of α-SMA, COL1a1, CD73, and A_1_R were increased (Figures 2C,D). These results suggested that CD73-A_1_R axis may play an important role in alcohol-related liver fibrosis, especially in the activation of HSCs.

### Knockout of CD73 Alleviates Alcohol-Related Liver Fibrosis

**FIGURE 3:**
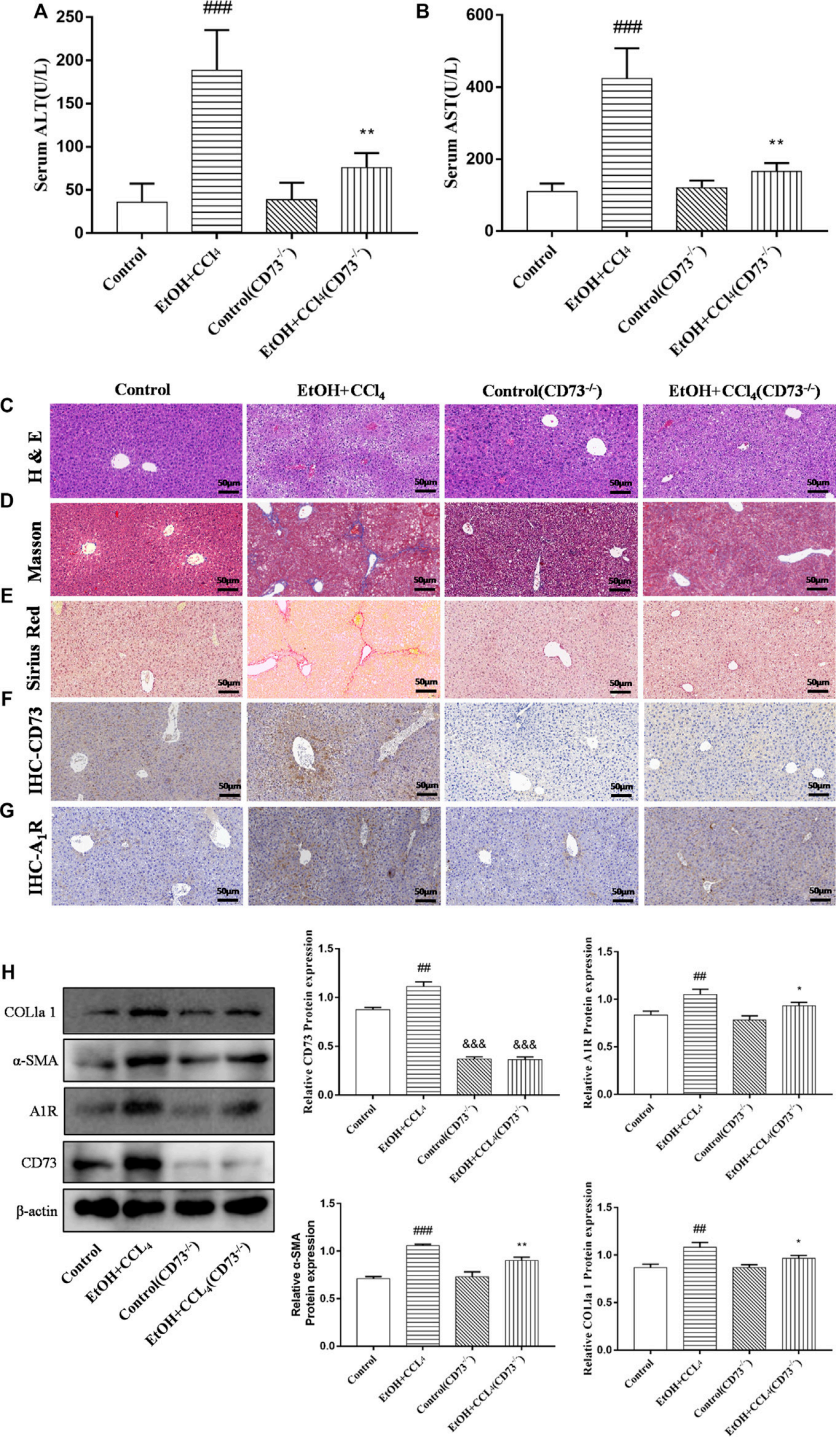
Knockout of CD73 alleviates ALF. **(A)** Serum ALT level. **(B)** Serum AST level. **(C)** Representative HE staining of liver sections (50 μm). **(D)** Representative Masson staining of liver sections (50 μm). **(E)** Representative Sirius Red staining of liver sections (50 μm). **(F)** Immunohistochemical (IHC) staining of CD73 in liver sections (50 μm). **(G)** Immunohistochemical (IHC) staining of A_1_R in liver sections (50 μm). **(H)** The protein levels of CD73, A_1_R, α-SMA and COL1a1 in the liver. ^*##*^ *p* < 0.01, ^*###*^ *p* < 0.001 *vs.* the control group. ^*^ *p* < 0.05, ^**^ *p* < 0.01 *vs.* the control (CD73^−/−^) group, ^*&&&*^ *p* < 0.001 *vs.* the control group.

To further explore the role of the CD73-A_1_R axis in ALF, CD73 knockout mice were used to establish ALF model. The results of immunohistochemistry and Western blot showed that CD73 was successfully knocked out in mice (Figures 3F,H). It can be seen from Figures 3A,B that knockdown of CD73 can reduce the high levels of ALT and AST induced by alcohol. The results of H & E staining, Masson staining, Sirius Red staining, immunohistochemistry and Western blot showed that knocking out CD73 had no significant effect on the liver tissue and various indicators of mice, and at the same time alleviated the disorder of liver cord, increased collagen deposition and α-SMA expression caused by alcohol (Figures 3C–E,H). At the same time, we found that A_1_R expression was slightly decreased in the control (CD73^−/−^) group, but there was no significant difference with the control group. Compared with the EtOH + CCl_4_ group, the A_1_R expression in the EtOH + CCl_4_ (CD73^−/−^) group was significantly reduced, suggesting that CD73 can affect ALF by regulating the expression of A_1_R (Figures 3G,H).

### Effect of Silencing CD73 on Hepatic Stellate Cell-T6 Cell Activation in Acetaldehyde-Induced Hepatic Stellate Cell-T6 Cell

**FIGURE 4:**
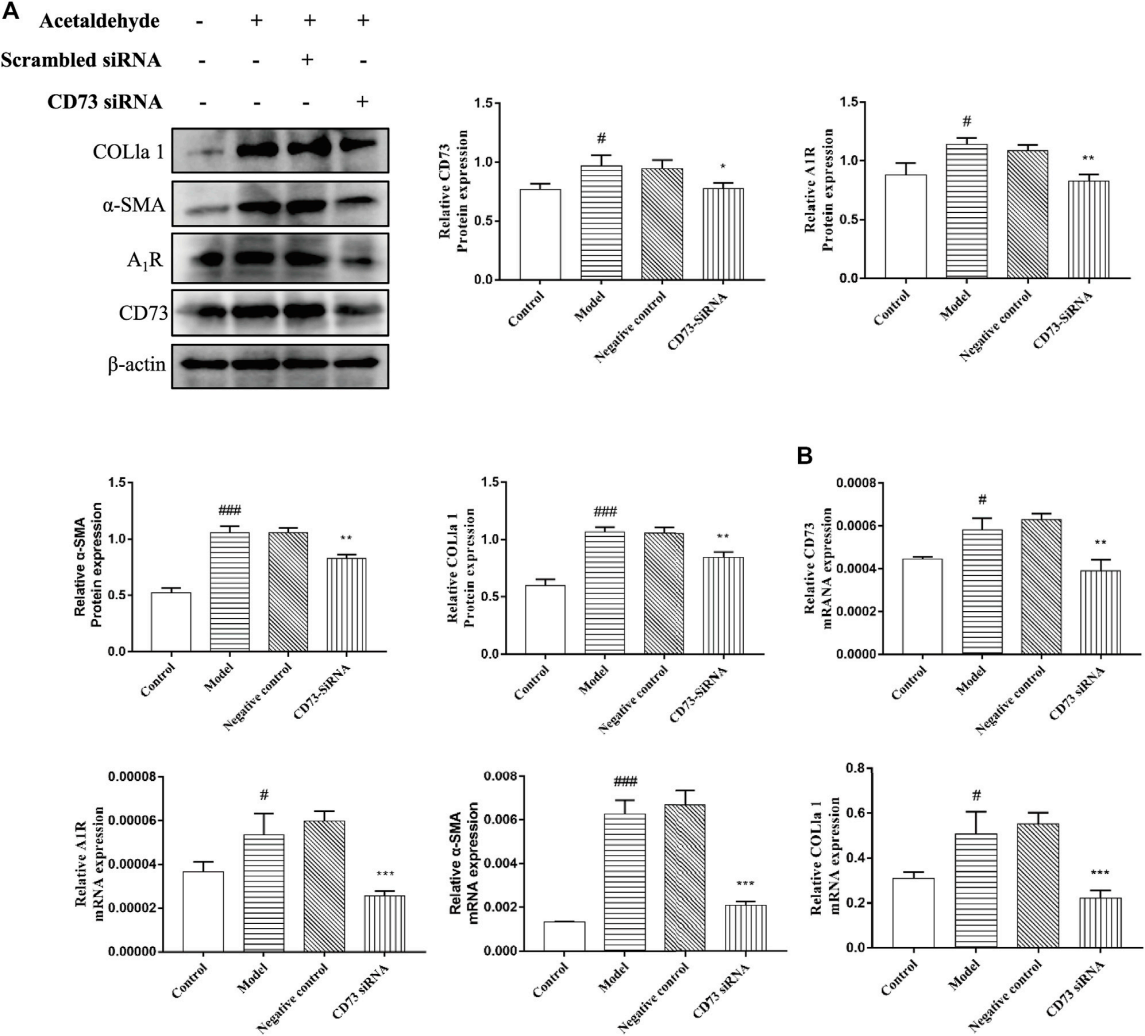
Effect of silencing CD73 on HSC-T6 cell activation in acetaldehyde-induced HSC-T6 cell. **(A)** The protein levels of CD73, A_1_R, α-SMA and COL1a1 in HSC-T6 cell. **(B)** The mRNA levels of CD73, A_1_R, α-SMA, and COL1a1 in HSC-T6 cell. ^*#*^ *p* < 0.05, ^*###*^ *p* < 0.001 *vs.* the control group. ^*^ *p* < 0.05, ^**^ *p* < 0.01, ^***^ *p* < 0.001 *vs.* the negative control group.

To further define the role of CD73 in ALF, CD73-siRNA was used to silence CD73 *in vitro*. As can be seen from Figures 4A,B, compared with the negative control group, the CD73 expression in the CD73 siRNA group was significantly reduced, indicating that CD73 was silenced successfully. At the same time, we found that silencing CD73 could reduce the increase of α-SMA and COL1a1 expression induced by acetaldehyde, which is consistent with the results obtained from the *in vivo* experiments (Figures 4A,B). In addition, we could also observe that the protein and mRNA levels of A_1_R increased after acetaldehyde stimulation, while the expression of A_1_R decreased when CD73 was inhibited (Figures 4A,B). This further suggests that CD73 may play an important role in ALF by acting on A_1_R.

### Effect of Silencing CD73 on Cell Apoptosis in Acetaldehyde-Induced Hepatic Stellate Cell-T6 Cell

**FIGURE 5:**
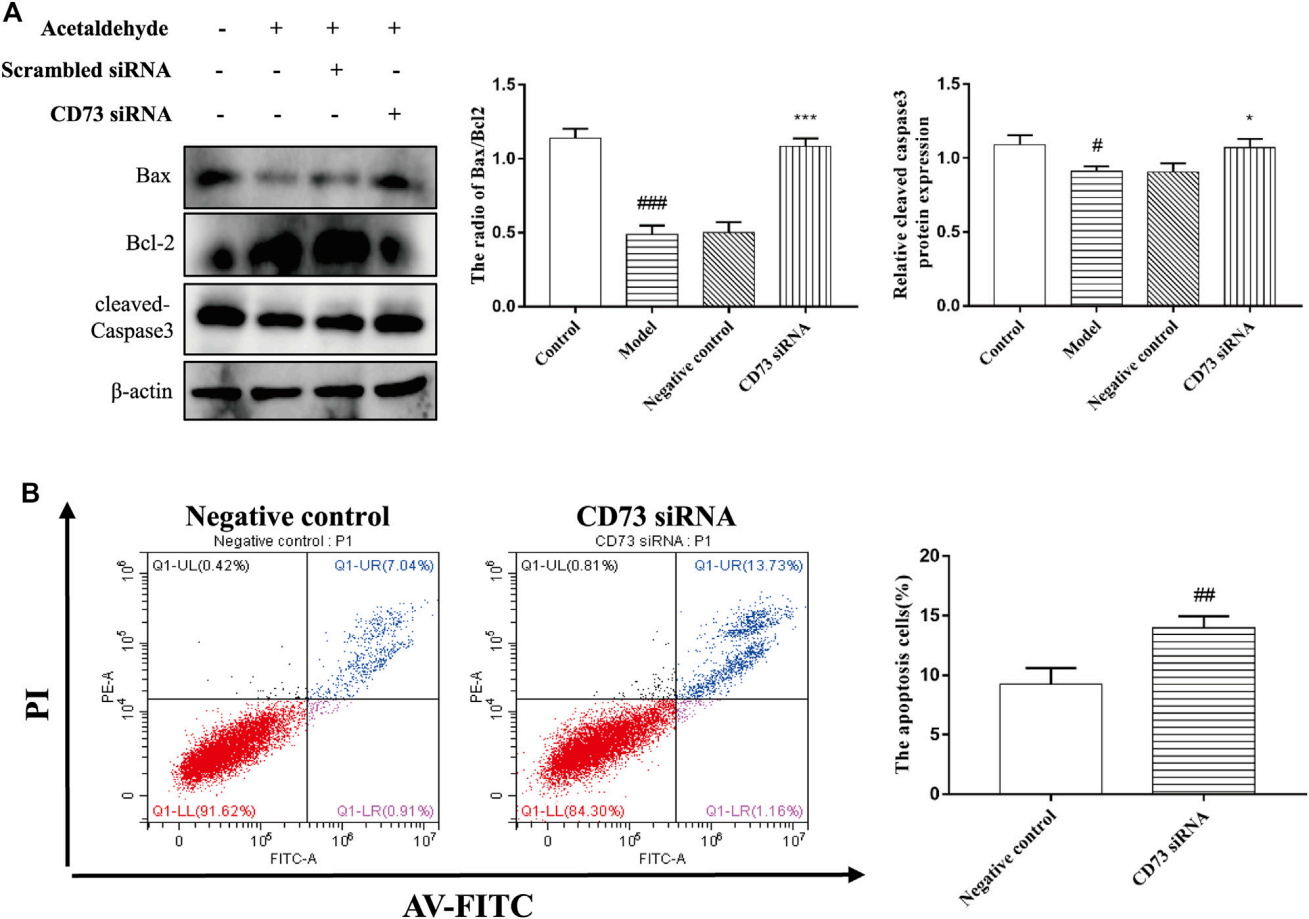
Effect of silencing CD73 on cell apoptosis in acetaldehyde-induced HSC-T6 cell. **(A)** Expression of Bax, Bcl-2, and cleaved caspase-3 in HSC-T6 cell. ^*#*^ *p* < 0.05, ^*###*^ *p* < 0.001 *vs.* control group. ^*^ *p* < 0.05, ^***^ *p* < 0.001 *vs.* the negative control group. **(B)** The apoptosis of HSC-T6 cell was determined by flow cytometry. ^*##*^ *p* < 0.01 *vs.* the negative control group.

To determine the role of CD73 in HSC-T6 cell apoptosis, Western blot and flow cytometry were used to detect apoptosis. From Figures 5A,B, it can be seen that the stimulation of acetaldehyde can inhibit the apoptosis of HSC-T6 cell, and the silencing of CD73 can significantly upregulate the ratio of Bax/Bcl-2 and the protein level of cleaved-Caspase3. The results of flow cytometry are consistent with it. These results suggest that silencing of CD73 can promote the apoptosis of HSC-T6 cell.

### Effect of Silencing A1 Receptor on Cell Activation in Acetaldehyde-Induced Hepatic Stellate Cell-T6 Cell

**FIGURE 6:**
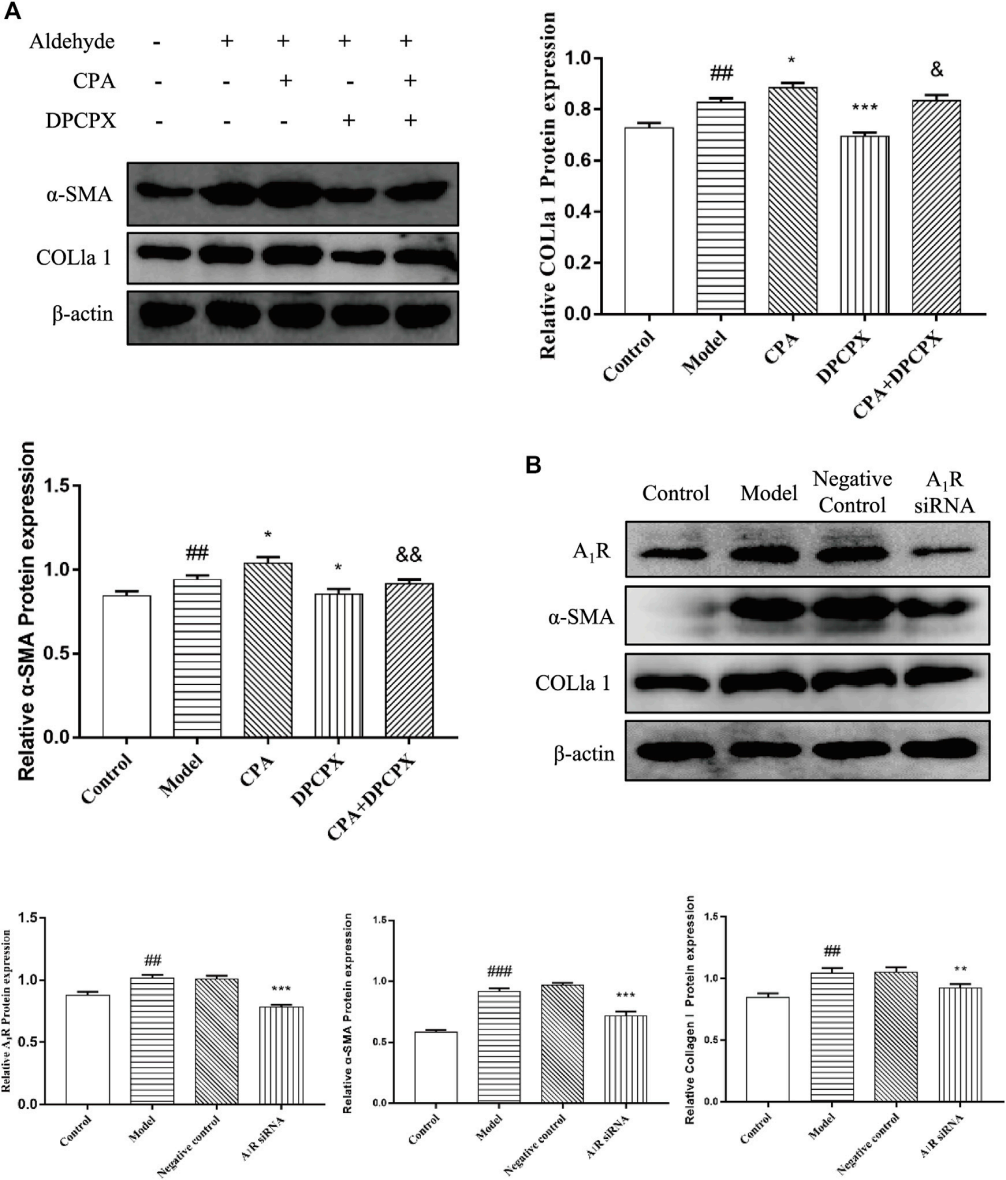
Effect of silencing A_1_R on cell activation in acetaldehyde-induced HSC-T6 cell. **(A)** The protein levels of α-SMA and COL1a1 in HSC-T6 cell. ^*##*^ *p* < 0.01 *vs.* the control group. ^*^ *p* < 0.05, ^***^ *p* < 0.001 *vs.* the model group. ^*&*^ *p* < 0.05, ^*&&*^ *p* < 0.01 *vs.* the CPA group. **(B)** The protein levels of A_1_R, α-SMA, and COL1a1 in HSC-T6 cell. ^*##*^ *p* < 0.01, ^*###*^ *p* < 0.001 *vs.* the control group. ^**^ *p* < 0.01, ^***^ *p* < 0.001 *vs.* the negative control group.

To elucidate whether A_1_R is involved in acetaldehyde-mediated fibrotic indicators (α-SMA and COL1a1) production, CPA and DPCPX were cultured with acetaldehyde-induced HSC-T6 cells. The results of real-time PCR and Western blot showed that DPCPX suppressed the levels of α-SMA and COL1a1, in contrast, CPA upregulated their expression. However, the effect above in the CPA + DPCPX group was obviously reversed (Figure 6A). Additionally, we transfected A_1_R siRNA into HSC-T6 cells with LipofectamineTM 2000. The results of Western blot showed that A_1_R siRNA effectively inhibited the levels of A_1_R, α-SMA and COL1a1 (Figure 6B). These results suggested that A_1_R is related to HSC-T6 cell activation.

### Effect of Silencing A1 Receptor on Cell Apoptosis in Acetaldehyde-Induced Hepatic Stellate Cell-T6 Cell

**FIGURE 7:**
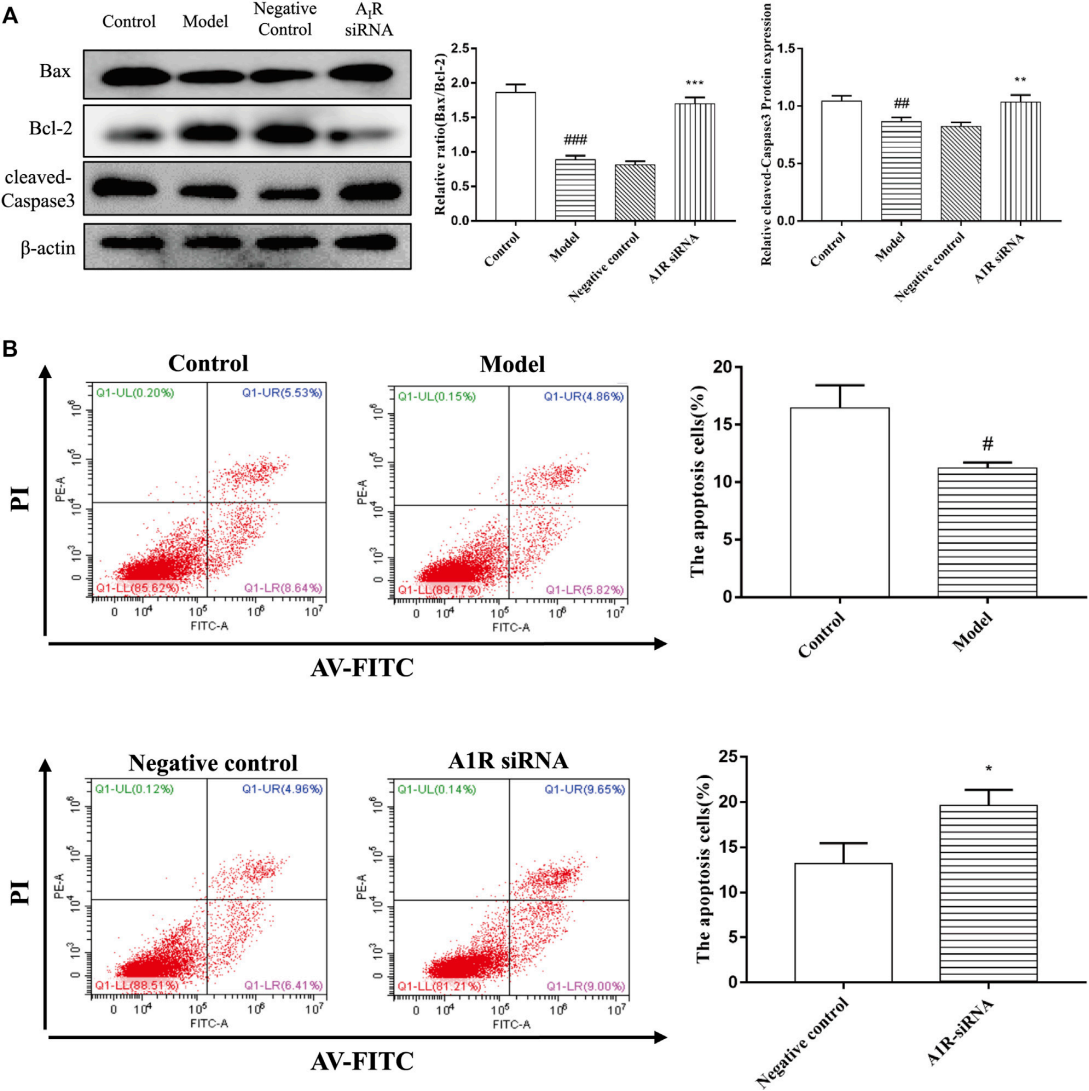
Effect of silencing A_1_R on cell apoptosis in acetaldehyde-induced HSC-T6 cell. **(A)** Expression of Bax, Bcl-2, and cleaved caspase-3 in HSC-T6 cell. **(B)** Effect of silencing A_1_R on the apoptosis of acetaldehyde-induced HSC-T6 cell was determined by flow cytometry. ^*#*^ *p* < 0.05, ^*##*^ *p* < 0.01, ^*###*^ *p* < 0.001 *vs.* the control group. ^*^ *p* < 0.05, ^**^ *p* < 0.01, ^***^ *p* < 0.001 *vs.* the negative control group.

To explore the effect of A_1_R on HSC-T6 cell apoptosis, we used siRNA to inhibit the expression of A_1_R and detect the related indicators. As shown in Figure 7A, compared with the negative control group, the ratio of Bax/Bcl-2 and the protein expression of cleaved caspase-3 in A_1_R siRNA group were upregulated. Flow cytometry results further confirmed the above results. Silencing A_1_R significantly increased the percentage of apoptosis in HSC-T6 cells (Figure 7B). In summary, these results suggest that A_1_R can inhibit the apoptosis of HSC-T6 cells.

### CD73 Positively Regulates PLC-IP_3_-Ca^2+^/DAG-PKC Signaling Pathway Activity in Acetaldehyde -Induced Hepatic Stellate Cell-T6 Cell

**FIGURE 8:**
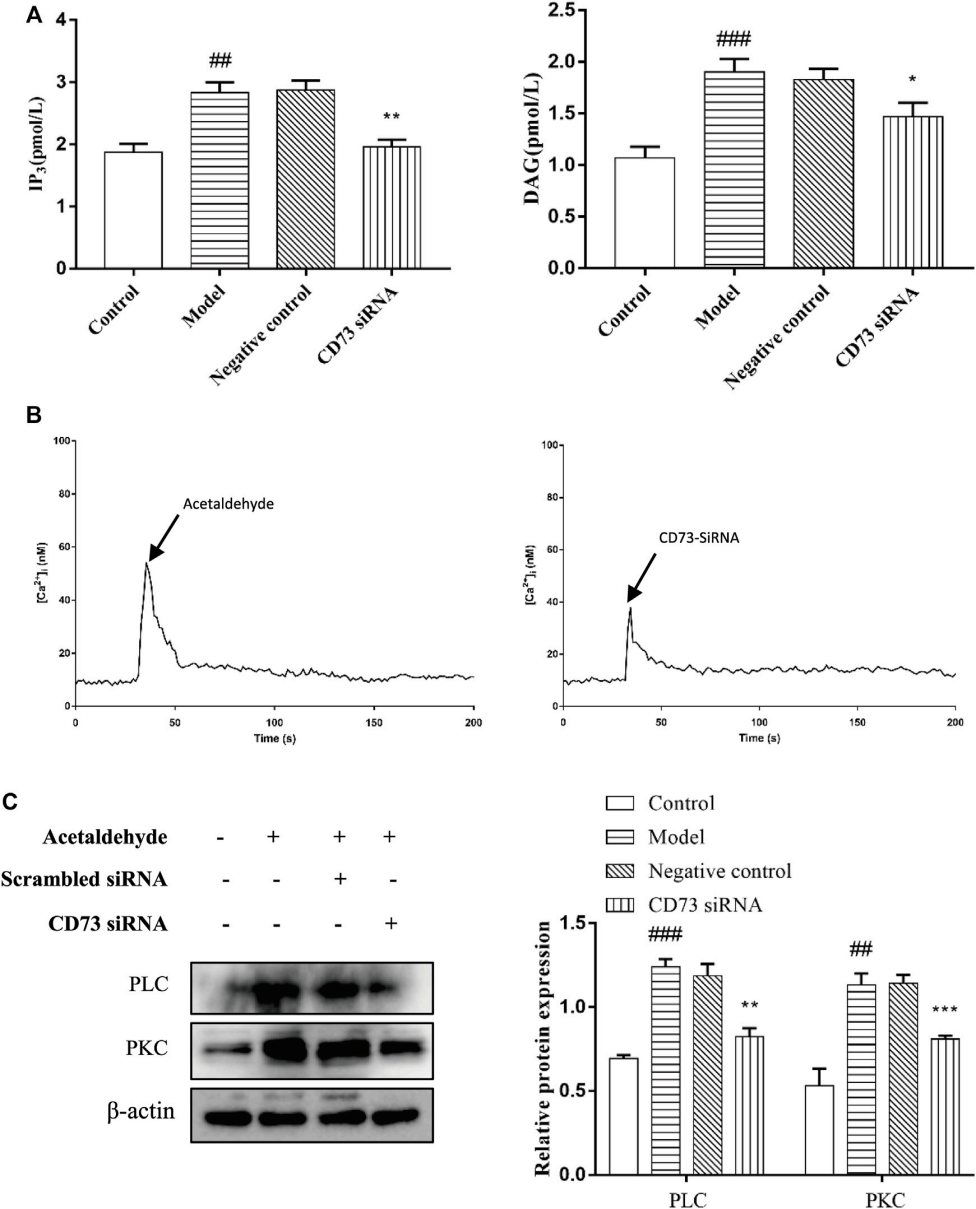
CD73 positively regulates PLC- IP_3_-Ca^2+^/DAG-PKC signaling pathway activity in acetaldehyde -induced HSC-T6 cell. **(A)** The content of IP_3_ and DAG were assessed by ELISA. **(B)** [Ca^2+^]_i_ concentration changes as visualized by Fluo-3-AM. **(C)** The protein levels of PLC and PKC in HSC-T6 cell. ^*##*^ *p* < 0.01, ^*###*^ *p* < 0.001 *vs.* the control group, ^*^ *p* < 0.05, ^**^ *p* < 0.01, ^***^ *p* < 0.001 *vs.* the negative control group.

There is evidence that PI signaling pathway plays a central role in cell proliferation, so we speculate that CD73/A_1_R axis may affect the activation and apoptosis of HSC-T6 cell through the PI signaling pathway. Hirsch et al. (2020) We transfected CD73 siRNA in acetaldehyde-stimulated HSC cell. The IP_3_ levels were measured with an Elisa kit, the protein expression levels of PLC and PKC were detected by Western blot and [Ca^2+^] concentration was monitored by confocal laser scanning microscope (CLSM). The results showed that compared with the control group, the expressions of PLC, IP_3_, DAG, and PKC in the model group were all increased. And the expressions of PLC, IP_3_, Ca^2+^, DAG, and PKC were all down-regulated after transfection of CD73 siRNA in acetaldehyde-induced HSC-T6 cells (Figures 8A–C). In conclusion, these results indicated that silencing CD73 blocks the PLC-IP_3_-Ca^2+^/DAG-PKC signaling pathway in ALF.

### A1 Receptor Positively Regulates PLC-IP_3_-Ca^2+^/DAG-PKC Signaling Pathway Activity in Acetaldehyde-Induced Hepatic Stellate Cell-T6 Cell

**FIGURE 9:**
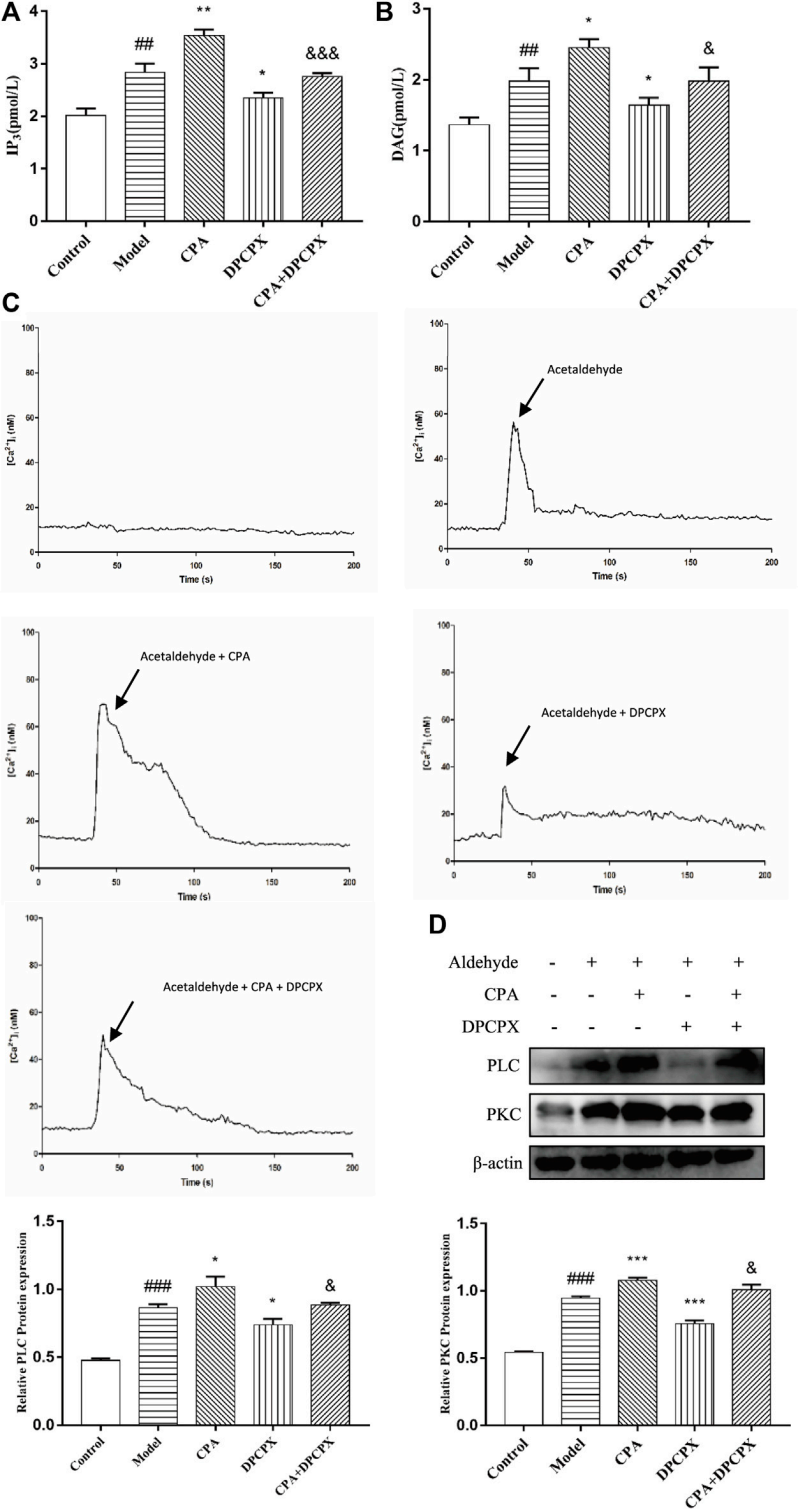
A_1_R positively regulates PLC- IP_3_-Ca^2+^/DAG-PKC signaling pathway activity in acetaldehyde-induced HSC-T6 cell. **(A)** The content of IP_3_ was assessed by ELISA. **(B)** The content of DAG was assessed by ELISA. **(C)** [Ca^2+^]_i_ concentration changes as visualized by Fluo-3-AM. **(D)** The protein levels of PLC and PKC in HSC-T6 cell. ^*##*^ *p* < 0.01, ^*###*^ *p* < 0.001 *vs.* the control group, ^*^ *p* < 0.05, ^**^ *p* < 0.01, ^***^ *p* < 0.001 *vs.* the model group. ^*&*^ *p* < 0.05, ^*&&&*^ *p* < 0.001 *vs.* the CPA group.

To explore whether A_1_R plays a role in ALF through PLC-IP3-Ca2^+^/DAG-PKC signaling pathway, the cells were divided into five groups: the control group, the model group, the CPA group (adenosine A_1_R agonist), the DPCPX group (adenosine A_1_R antagonist) and the CPA + DPCPX group. The results show that the levels of IP_3_, DAG, PLC, PKC, and [Ca^2+^] concentration in model group were enhanced obviously at 48 h after acetaldehyde stimulation while significantly increased after CPA treatment but reduced after DPCPX treatment. However, the results of the CPA + DPCPX group were significantly reversed (Figures 9A–D). The expression levels of α-SMA and COL1a1 were detected by real-time quantitative PCR and Western blot. The result trend is consistent with the above indicators (Figure 6A). Taken together, the results indicated that the A_1_R might regulate the progression of ALF through the PLC-IP_3_-Ca^2+^/DAG-PKC signaling pathway.

### Effect of the PLC-IP_3_-Ca^2+^/DAG-PKC Signaling Pathway in Acetaldehyde-Induced Hepatic Stellate Cell-T6 Cell

**FIGURE 10:**
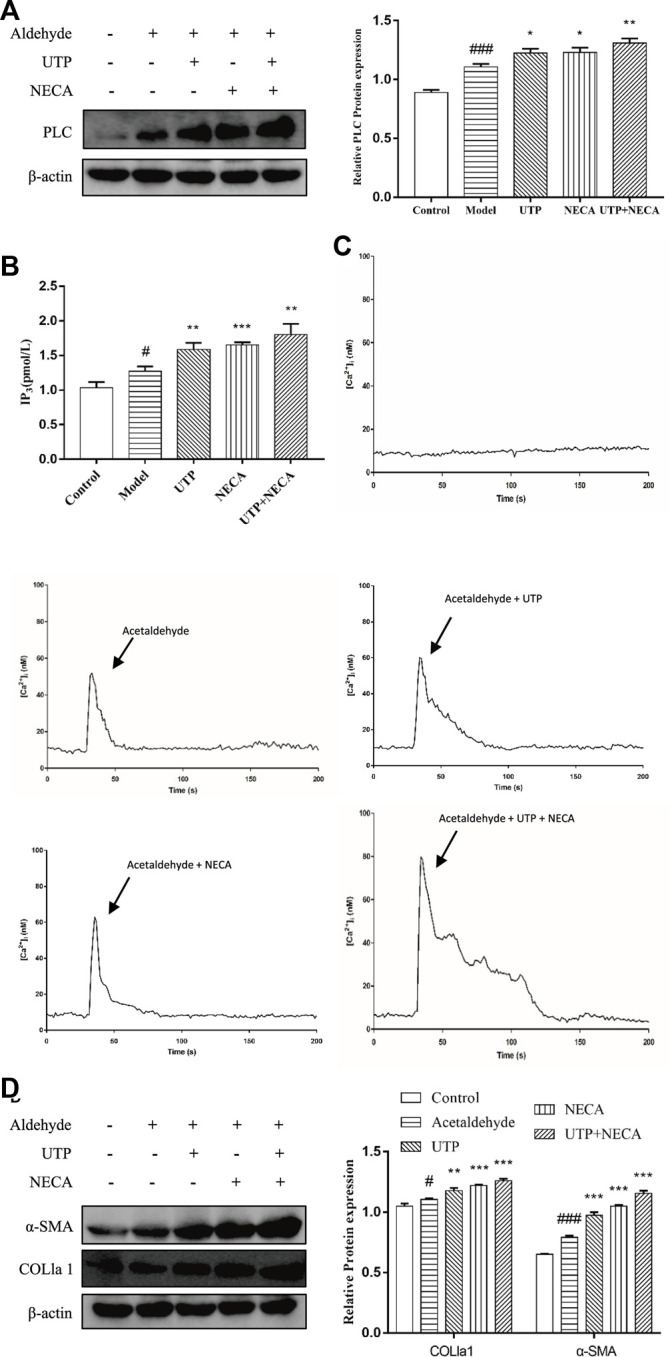
Effect of the PLC- IP_3_-Ca^2+^/DAG-PKC signaling pathway in acetaldehyde-induced HSC-T6 cell. **(A)** The protein levels of PLC in HSC-T6 cell. **(B)** The content of IP_3_ was assessed by ELISA. **(C)** [Ca^2+^]_i_ concentration changes as visualized by Fluo-3-AM. **(D)** The protein levels of α-SMA and COL1a1 in HSC-T6 cell. ^*#*^ *p* < 0.05, ^*###*^ *p* < 0.001 *vs.* the control group, ^*^ *p* < 0.05, ^**^ *p* < 0.01, ^***^ *p* < 0.001 *vs.* the model group.

**FIGURE 11:**
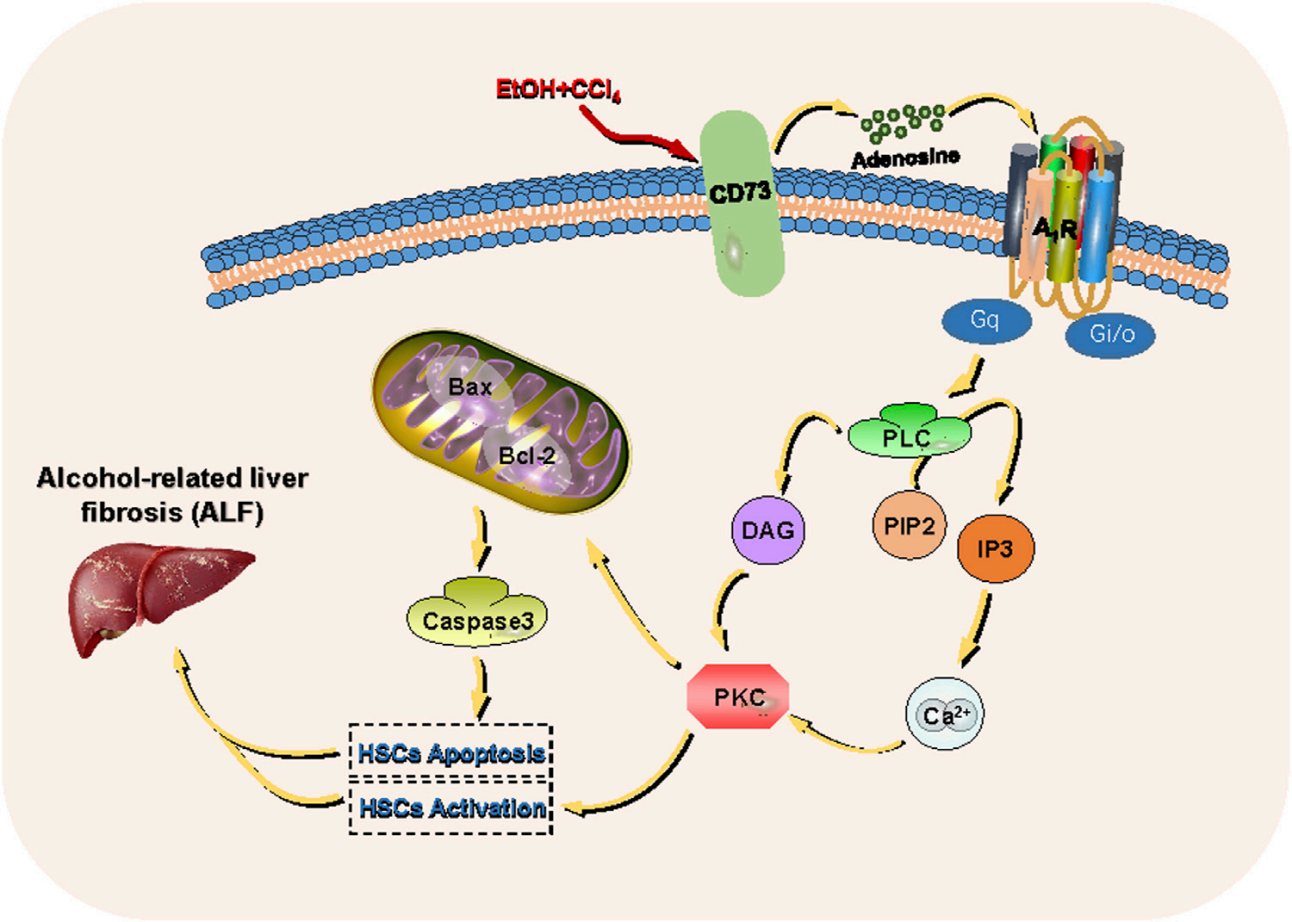
CD73- A_1_R axis regulates the activation and apoptosis of HSCs through the PLC-IP_3_-Ca^2+^/DAG-PKC signaling pathway.

To determine whether the PLC-IP_3_-Ca^2+^/DAG-PKC signaling pathway participates in ALF, HSC-T6 cell incubated for 24 h and HSCs were divided into five groups: the control group, the model group, the UTP group (precursor of IP_3_), the NECA group (nonselective adenosine receptor agonist) and the UTP + NECA group. Acetaldehyde(200 µM) was added to each group for 48 h to establish an alcohol-related liver fibrosis model, except for the control group, and then UTP and NECA were added into each group respectively. The results showed that compared with the model group, the expression of IP_3_ and key signaling molecules, such as PLC and [Ca^2+^], was significantly increased in the UTP and NECA groups (Figures 10A–C). The protein levels of α-SMA and COL1a1 were also upregulated in the UTP + EPCA groups (Figure 10D). UTP can significantly enhance the effect of NECA. These results indicated that the PI signaling pathway was correlated with HSC activation and positively regulated the expression of α-SMA and COL1a1. In conclusion, CD73-A_1_R axis can regulate the activation and apoptosis of HSC-T6 cell through the PLC-IP_3_-Ca^2+^/DAG-PKC signaling pathway (Figure 11).

## Discussion and Conclusion

In the past 30 years, with the explosive growth of Chinese economy and the increase in alcohol consumption, the incidence rate of ALD has also increased. Alcohol-related liver injury is caused by oxidative alcoholic metabolites such as acetaldehyde and reactive oxygen species (ROS). Lu and Cederbaum (2008); Mashiko et al. (2010) These metabolites can cause aberrant oxidative phosphorylation and mitochondrial DNA damage. The subsequent lipid peroxidation products produced by accumulated oxygen free radicals and lipids further aggravates oxidative damage in ALD. Bradford et al. (2005); Lu et al. (2010) Liver fibrosis is a dynamic process of fibrinogenesis and fibrinolysis. Although it is a prerequisite for cirrhosis progression, it is also vital in the reversible recovery of ALD. Louvet and Mathurin (2015); Mehal and To (2016); Smith et al. (2019) HSCs are quiescent cells in the perisinusoidal space of the liver. After stimulation with ethanol and its metabolite acetaldehyde, HSCs become activated and proliferate. Activated HSCs are the main source of COL1a1 in the liver. Therefore, the inactivation and depletion of HSCs is very important for the treatment of ALF. Zhang et al. (2016).

Recently several studies have shown that CD73 plays a key role in tumorigenesis, immune escape, and immunotherapy. Ghalamfarsa et al. (2018); Chen et al. (2020) In a previous study by our group, suramin was used *in vivo* to inhibit CD73 in mice, inhibition of CD73 was shown to alleviate ALF. In this study, CD73 knockout mice were used to further verify the role of CD73 in ALF and to explore the effects on the mechanisms involved.

As an important hydrolase in the purine signaling pathway, CD73 generates adenosine by hydrolyzing ATP, which plays a role in various diseases by acting on adenosine receptors. Adenosine and its receptors are important in the pathogenesis of fibrotic diseases. Due to different pathophysiological conditions, the differences in the structure and function of AR target cells results in changes in the activation state. Changes in the activation state activate different downstream signaling pathways, leading to diverse regulatory effects of fibrotic disease. For example, activated A_1_R can reduce injury in lung ischemia-reperfusion, and it can also participate in lung fibrotic disease as an inflammatory cytokine. Ajamieh et al. (2008); Kalk et al. (2009) However, the role and mechanism of the CD73/A_1_R axis in alcohol-related liver fibrosis requires further investigation. Our previous studies have shown that CD73 and A_1_R can promote HSC activation, but the specific mechanism and its effect on apoptosis of HSC remain unclear. Yang et al. (2015).

Ramirez et al. performed a long-term (eight-weeks) ethanol-plus-multiple binges of ethanol to establish a mouse model of alcohol-related liver fibrosis, resulting in more liver fibrosis in middle-aged mice than in young mice. Currently, there is no mature mouse model of alcohol-related liver fibrosis, however available literature indicates that mice liver fibrosis models established with alcohol and carbon tetrachloride (CCl_4_) can mimic a human alcohol-related liver fibrosis model. Brol et al. (2019) Finally, an alcohol-related liver fibrosis model in mice was successfully established after eight-weeks ethanol-plus-multiple binges of ethanol combined with CCl_4_. In this study, the expression of CD73 and A_1_R was shown to be elevated in the liver tissues of model mice and in acetaldehyde-treated HSC-T6 cells. CD73/A_1_R deficiency or inhibition significantly reduced the degree of fibrosis and promoted apoptosis in HSC-T6 cells.

Alcohol induces HSCs to release numerous adenosines which activates ARs coupled with G-proteins to turn on adenylate cyclase. Diehl et al. (2010) The ALF, cAMP, and PI signaling pathways mediated by G protein-coupled receptors are the main cellular targets of alcohol effects, with key molecules of the PI signaling pathway being affected. Both DAG and PKC are important proponents in cell proliferation. Gary et al. (2001); Yang et al. (2002); Nitti et al. (2008) Reports have shown that A_1_R is related to cell proliferation in renal cell carcinoma. Zhou et al. (2017) Stimulus-induced activation of PLC which cleaves PLC to hydrolyze phosphatidylinositol bisphosphate resulting in the production of DAG and IP_3._ This leads to protein PKC activation or IP_3_ receptor-dependent Ca^2+^ release. PKC is activated, thereby phosphorylating its target protein to exert biological effects. Liu et al. (2020) Therefore, we speculated that the CD73-A_1_R axis may regulate HSC activation and apoptosis *via* the PLC-IP_3_-Ca^2+^/DAG-PKC signaling pathway.

The possibility of an AR-coupled PI pathway using the UTP and NECA in the ALF HSC model was also discussed. This study suggested that the PLC-IP_3_-Ca^2+^/DAG-PKC signaling pathway is present in HSCs and that the PLC-IP_3_-Ca^2+^/DAG-PKC signaling pathway has a regulatory role in the activation and proliferation of HSCs. To further investigate the role of the CD73-A_1_R axis in the ALF HSC model *via* the PLC-IP_3_-Ca^2+^/DAG-PKC pathway, CD73 and A_1_R modulators were used in an *in vitro* model and the expression of the downstream pathways was evaluated. The results showed that CD73 could alter the proteolytic activity of PLC by acting on A_1_R to influence the contents of IP_3_ and DAG, thereby controlling the release of Ca^2+^ and PKC activity. Thus, we believe that the CD73-A_1_R axis can regulate HSC-T6 cell activation and apoptosis through the PLC-IP_3_-Ca^2+^/DAG-PKC signaling pathway.

The intracellular pathway, which is mediated by G protein-coupled receptors, constitutes a complex regulatory network. For example, in cells such as platelets or lymphocytes, the PLC-PKC pathway is often antagonized by cAMP, while in other cell types, such as rat granulosa cells, inhibition of PLC or PKC can activate the cAMP pathway. Mosenden and Taskén (2011); Nermer et al. (2018); Rostami et al. (2019) Studies have shown that there is not only positive crosstalk but also negative crosstalk between PLC-PKC and cAMP-PKA. Nalli et al. (2014); Rahamim Ben-Navi et al. (2016) Our previous study showed that both A_1_R and A_2A_R regulated the cAMP-PKA-CREB signaling pathway with opposite effects. Antagonists of A_1_R can promote the activation of the cAMP pathway and induce the proliferation of HSCs. Yang et al. (2015) We suggest that there is crosstalk between PLC-PKC and cAMP-PKA in HSCs and that there may be a relationship between the pathways mediated by A_1_R and A_2A_R. Future research should address these issues.

In summary, our findings suggest that the CD73-A_1_R axis is critical for cell activation and apoptosis through the PLC-IP_3_-Ca^2+^/DAG-PKC signaling pathway in acetaldehyde-induced HSC-T6 cells. These results imply that the CD73-A_1_R axis may be a promising target for the suppression of ALF and provide evidence for further exploration of the role of the purine signaling pathway in ALD.

## Data Availability

The original contributions presented in the study are included in the article/Supplementary Material, further inquiries can be directed to the corresponding authors.
