# Supplementary figures and images for: CD73-Adenosine A1R Axis Regulates the Activation and Apoptosis of Hepatic Stellate Cells Through the PLC-IP3-Ca2+/DAG-PKC Signaling Pathway
Source: Front Pharmacol. 2022 Jun 16;13:922885. doi: 10.3389/fphar.2022.922885 (PMC9245432; doi:10.3389/fphar.2022.922885)

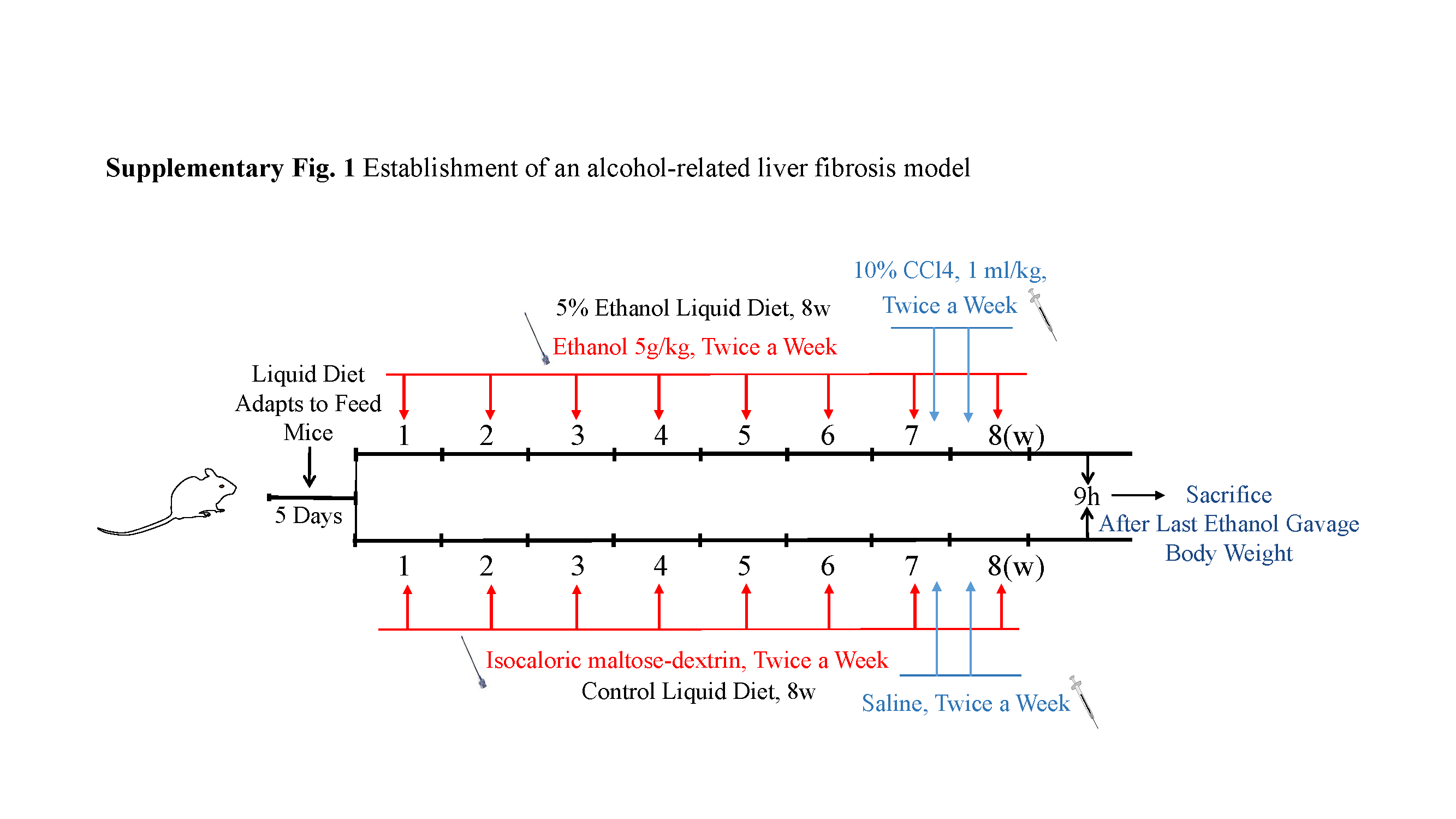

Supplement: Supplementary file 1 [file Image1.TIFF]
